# Different scales of gene duplications occurring at different times have jointly shaped the NBS-LRR genes in *Prunus* species

**DOI:** 10.1007/s00438-021-01849-z

**Published:** 2022-01-15

**Authors:** Yan Zhong, Zhao Chen, Zong-Ming Cheng

**Affiliations:** College of Horticulture, State Key Laboratory of Crop Genetics and Germplasm Enhancement, Nanjing Agricultural University, Nanjing, 210095 China

**Keywords:** NBS-LRR genes, *Prunus* species, Disease resistance genes, Species-specific duplications, Lineage-specific duplications

## Abstract

In this study, genome-wide identification, phylogenetic relationships, duplication time and selective pressure of the NBS-LRR genes, an important group of plant disease-resistance genes (*R* genes), were performed to uncover their genetic evolutionary patterns in the six *Prunus* species. A total of 1946 NBS-LRR genes were identified; specifically, 589, 361, 284, 281, 318, and 113 were identified in *Prunus yedoensis*, *P. domestica*, *P. avium*, *P. dulcis*, *P. persica* and *P. yedoensis* var. *nudiflora*, respectively. Two NBS-LRR gene subclasses, TIR-NBS-LRR (TNL) and non-TIR-NBS-LRR (non-TNL), were also discovered. In total, 435 TNL and 1511 non-TNL genes were identified and could be classified into 30/55/75 and 103/158/191 multi-gene families, respectively, according to three different criteria. Higher *Ks* and *Ka/Ks* values were detected in TNL gene families than in non-TNL gene families. These results indicated that the TNL genes had more members involved in relatively ancient duplications and were affected by stronger selection pressure than the non-TNL genes. In general, the NBS-LRR genes were shaped by species-specific duplications, and lineage-specific duplications occurred at recent and relatively ancient periods among the six *Prunus* species. Therefore, different duplicated copies of NBS-LRRs can resist specific pathogens and will provide an *R*-gene library for resistance breeding in *Prunus* species.

**Supplementary Information:**

The online version contains supplementary material available at 10.1007/s00438-021-01849-z.

## Introduction

*Prunus* species are widely cultivated around the world for their economic and ornamental value. Such species include the cherry plum (*P*. *cerasifera* Ehrh.), sour cherry (*P. cerasus* L.), plums (*P. domestica* L. and *P. insititia* L.), invasive black cherry (*P. serotina* Ehrh.), and almond (*P. communis* Archang.) (Cici and Van Acker 2010). However, these *Prunus* trees can be infected by various pathogens. Thus, it is necessary to conduct a genetic investigation of the NBS-LRR genes in *Prunus* species. Based on the release of the whole-genome sequences of six *Prunus* species, *P. yedoensis*, *P. domestica*, *P. avium*, *P. dulcis*, *P. persica* and *P. yedoensis* var. *nudiflora*, a genome-wide identification and genetic evolutionary analysis of the NBS-LRR genes in these species was performed in this study.

Plants are constantly subjected to varying environmental conditions and stresses during their growth and development stages. In particular, plants are frequently attacked by a series of bacteria, nematodes, fungi, and insects (Dangl and Jones 2001). As important constituents of the plant innate immune system, disease-resistance genes (*R* genes) encode specific receptors that recognize pathogenic avirulence *(Avr*) genes (Keen 1990; Dangl and Jones 2001; Jones and Dangl 2006). A large proportion of plant *R* genes are called NBS-LRR genes and encode a conserved nucleotide-binding site (NBS) domain and leucine-rich repeat (LRR) motifs (McHale et al. 2006). The N-terminal structures of NBS-LRR proteins contribute to pathogen recognition and downstream signal transduction (Maekawa et al. 2011). According to the characteristics of the N-terminal domain, NBS-LRR proteins are divided into two subgroups. Proteins in one subgroup have a Toll/interleukin-1 receptor (TIR) domain at the N-terminal region (TIR-NBS-LRR proteins), and proteins of the other subgroup contain a coiled-coil (CC) domain, resistance to powdery mildew8 (RPW8) domain, or another type of domain (X) (non-TIR-NBS-LRR proteins) (Xiao et al. 2001; Meyers et al. 2003; Collier et al. 2011).

Plant NBS-LRR proteins are abundant in quantity and ancient in origin. The main function of NBS-LRR genes is resistance to disease by way of pathogen recognition (Dangl and Jones 2001; Meyers et al. 2003). NBS-LRR genes exist widely and have been detected in many different plant species, such as *Arabidopsis thaliana*, rice (*Oryza sativa*), cassava (*Manihot esculenta*), *Medicago* species, and potato (*Solanum tuberosum*) (Dangl and Jones 2001; Monosi et al. 2004; Ameline-Torregrosa et al. 2008; Lozano et al. 2012, 2015). However, variations in the copy numbers of NBS-LRR genes have been commonly found among different species. For example, there are only 50 NBS-LRR genes in papaya but 1015 NBS-LRRs in the apple genome (Porter et al. 2009; Arya et al. 2014). A previous study assumed that all plant *R* genes originate from a common ancestor and that gene duplications have led to variations in the copy numbers of NBS-LRR genes among different species (Meyers et al. 2005). Therefore, the expansion of NBS-LRR genes in specific species may help the plant adapt to rapid changes in the genome of the specific pathogens that harm it (Li et al. 2010). For example, the NBS-LRR genes were shaped by gene amplification in kiwifruit (*Actinidia chinensis*), in which some of them exhibited a positive response to bacterial canker disease infection (Wang et al. 2020); the *Pi2*/*Pi9* genes encoding NBS-LRR proteins, located in a duplicated cluster of rice, showed broad-spectrum resistance against rice blast (Zhou et al. 2006; Zhu et al. 2012).

*Prunus* contains approximately 250 plant species and consists of five subgenera (Mace 1940; Robertson et al. 2004; Baek et al. 2018). This study was supported by relatively good whole-genome sequencing data of the six *Prunus* species. In *P. yedoensis*, an artificial cross from *P. spachiana* and *P. speciosa* (or closely related species), the total sequences with 192-fold genome coverage were assembled into 690.1 Mb and identified 95,076 high-confidence annotated genes (Shirasawa et al. 2019). For *P. yedoensis* var. *nudiflora*, also named *Cerasus* x *nudiflora*, a natural hybrid from *P. pendula f. ascendens* and *P. jamasakura*, which is a distinguishable species of *P. yedoensis* (Jung and Oh 2005; Roh et al. 2007; Baek et al. 2018). In the heterozygous genome of *P. yedoensis* var. *nudiflora*, 323.8 Mb assembled draft genome sequences with annotation of 41,294 protein-encoding genes were obtained based on 73-fold coverage reads capturing 126% of the estimated haploid genome (Baek et al. 2018). In *P. avium* (sweet cherry), 272.4 Mb of assembly sequences occupied 77.8% of the estimated genome (352.9 Mb) (Shirasawa et al. 2017). In addition, the total read coverage of *P. domestica* was 210-fold, in which 130,866 genes were annotated as predicted (Genome Database for Rosaceae, GDR, https://www.rosaceae.org/). The assembly completion rate of *P. dulcis* exceeds 96% according to the 285-fold coverage reads (GDR, https://www.rosaceae.org/). Finally, for the genome sequences of *P. persica*, higher accuracy and more mapped sequences were performed compared with the high-quality first assembly version (Verde et al. 2017). In total, 1946 NBS-LRR genes were identified among the six *Prunus* species. Subsequently, the duplication time, selection pressure, and phylogenetic relationships of the NBS-LRR genes were examined here. It was found that both species-specific duplications and lineage-specific duplications jointly contributed to the expansion of NBS-LRR genes in the six *Prunus* species. In addition, the TNL and non-TNL genes showed distinct evolutionary patterns.

## Materials and methods

### Identification of NBS-LRR genes

To detect the NBS-LRR genes in the six *Prunus* species, the whole-genome sequences of *P. yedoensis*, *P. domestica*, *P. avium*, *P. dulcis*, *P. persica* and *P. yedoensis* var. *nudiflora* were downloaded from the site (GDR, https://www.rosaceae.org/). The NB-ARC domain was searched in the annotated proteins of the whole genomes of all six species using local InterProScan with default settings. Genes encoding NB-ARC domains were considered NBS-encoding candidate genes for further LRR detection. SMART (a Simple Modular Architecture Research Tool; http://smart.embl-heidelberg.de/) was used to examine whether the NBS-encoding genes encoded the LRR motifs. NLR-parser analysis (https://github.com/steuernb/NLR-Parser) was also performed to enhance the accuracy of LRR motif annotation. Subsequently, Pfam analysis was used to determine whether these NBS-LRR genes encoded TIR, RPW8, or other domains. The CC domain was confirmed using the COILS databases (http://embnet.vital-it.ch/software/COILS_form.html).

### Classification of the NBS-LRR gene families

The nucleotide coding sequences (CDSs) of the TNL and non-TNL genes in the six *Prunus* species were processed using all-versus-all BLASTN searches with an *E*-value of 1.0 (Zhong et al. 2018). Based on the BLAST results, two parameters, the coverage and identity between sequences, both larger than 70%, were used to classify the TNL and non-TNL genes into gene families. Subsequently, to analyze the relatively recent duplications of NBS-LRR genes in the six *Prunus* species, both the coverage and identity criteria were increased to stricter standards of > 80% and > 90% (Yang et al. 2008; Zhong et al. 2015).

All of the CDSs of the NBS-LRR genes were aligned with the guidance of amino acid sequence alignments using Clustalw2.0 with default settings (Larkin et al. 2007). Subsequently, MEGA X (Kumar et al. 2018) was employed to calculate synonymous substitutions (*Ks*), nonsynonymous substitutions (*Ka*), and the ratios of nonsynonymous to synonymous substitutions (*Ka*/*Ks*) in each gene family of TNLs and non-TNLs. The mutation rate of peach was 9.48 × 10^–9^ point mutations per site per generation (Xie et al. 2016), experiencing less than 3 years per generation (Dennis 2009). The duplication time of the NBS-LRR genes was calculated based on the formula *T* = *Ks*/9.48 × 10–9/3.

In addition, the nucleotide diversity (*Pi* value) of each TNL and non-TNL gene family was determined by MEGA X (Kumar et al. 2018), and sequence exchange events were conducted by GENECONV 1.81 (http://www.math.wustl.edu/sawyer/geneconv/) using the default setting with 10,000 permutations (*P* value < 0.05) (Chen et al. 2010).

### Phylogenetic tree of NBS-LRR genes

The nucleotide sequences of the NB-ARC domains of the obtained TNL and non-TNL genes were aligned using the MUSCLE (Multiple Sequence Comparison by Log-Expectation) program with MEGA X (Kumar et al. 2018). Subsequently, the alignments were used to construct two maximum likelihood (ML) phylogenetic trees of TNLs and non-TNLs with MEGA X (Kumar et al. 2018) using the Tamura-Nei model with 1000 replicates. Species-specific duplications were defined as NBS-LRR duplications that appeared in only one *Prunus* species, and lineage-specific duplications occurred in two or more *Prunus* species.

### Differentially expressed NBS-LRR genes in peach after aphid infection

The RNA-seq data of two *P. persica* lines, resistant individual R36 and susceptible individual S38, were detected after infection with the green peach aphid (GPA) after 0, 3, 6, 9, 12, 24, 48 and 72 h (Niu et al. 2018a). NBS-LRRs belonging to differentially expressed genes (DEGs), with the criteria of |logFC|≥ 2 (*P* < 0.05) and FDR < 0.05, and their FPKM values (fragments per kilobase of transcript per million mapped fragments) were screened out to generate heatmaps in R36 and S38 by R script.

## Results

### NBS-LRR genes in six *Prunus* species

Using BLAST searches, a total of 1946 NBS-LRR genes were detected in the six *Prunus* genomes. Of the six species, *P. yedoensis* possessed the largest number of NBS-LRR genes at 589. For the other five species, *P. domestica*, *P. avium*, *P. dulcis*, *P. persica* and *P. yedoensis* var. *nudiflora*, 361, 284, 281, 318, and 113 NBS-LRR genes were identified, respectively (Table 1). Because the assembly genome of *P. yedoensis* var. *nudiflora* was based on homozygous reads possessing half the size of *P. yedoensis*, leading to a distinct difference in NBS-LRR gene numbers between the two species (Baek et al. 2018). Among the NBS-LRR genes, the two subgroups of genes, TNLs and non-TNLs, could be classified based on their N-terminal domains (Zhong et al. 2015). In total, 435 TNL genes and 1511 non-TNL genes were identified in the six species. Specifically, 162, 3, 76, 87, 105, and 2 TNL genes and 427, 358, 208, 194, 213, and 111 non-TNL genes were identified in *P. yedoensis*, *P. domestica*, *P. avium*, *P. dulcis*, *P. persica* and *P. yedoensis* var. *nudiflora*, respectively*.* The numbers of non-TNL genes were significantly greater than the numbers of TNL genes among the six species (*t* test,* P* < 0.05).

**Table 1:** Numbers of NBS-LRR genes in six *Prunus* genomes

Predicted protein domains	Letter code	*P. yedoensis*	*P. domestica*	*P. avium*	*P. dulcis*	*P. persica*	*P. yedoensis var. nudiflora*	Total
NBS-LRR		589	361	284	281	318	113	1946
TIR-NBS-LRR	TNL	162	3	76	87	105	2	435
Non-TIR-NBS-LRR	Non-TNL	427	358	208	194	213	111	1511
CC-NBS-LRR	CNL	253	186	103	114	128	53	837
CC-NBS-LRR'	CNL'	251	186	103	106	128	53	827
RPW8-CC-NBS-LRR	RPW8-CNL	2	0	0	8	0	0	10
X-NBS-LRR	XNL	174	172	105	80	85	58	674
X-NBS-LRR'	XNL'	173	169	101	79	84	56	662
RPW8-X-NBS-LRR	RPW8-XNL	1	3	4	1	1	2	12

According to the existence of different N-terminal protein domains, the non-TNL genes of the six *Prunus* species were classified into two subtypes: 837 CC-NBS-LRR (CNL) genes and 674 X-NBS-LRR (XNL) genes. The CNL subtype contained 827 CC-NBS-LRR’ (CNL’) and 10 RPW8-CC-NBS-LRR (RPW8-CNL) genes. The XNL subtype incorporated 662 X-NBS-LRR’ (XNL’) and 12 RPW8-X-NBS-LRR (RPW8-XNL) genes (Table 1). The RPW8-CNL and RPW8-XNL genes, which contained an N-terminal RPW8 domain, were found in all six of the surveyed species.

### NBS-LRR multi-genes in six *Prunus* species

To detect the multi-gene families of the six *Prunus* species, gene families were defined based on two criteria: (1) a coverage greater than 70% and (2) an identity between sequences larger than 70%. A total of 409 TNL multi-genes and 1357 non-TNL multi-genes were detected in 30 TNL gene families and 103 non-TNL gene families, respectively (Table 2). The proportion of TNL genes in multi-gene families (94.02%) was greater than that of non-TNL genes in multi-gene families (89.81%). Overall, 90.75% (1766/1946) of all the NBS-LRR genes were contained in multi-gene families (Table 2). Although different numbers of multi-genes were detected in each species, distinctly similar proportions of multi-genes were found in both TNL and non-TNL gene families.

**Table 2:** Classification of NBS-LRRs in genomes of six *Prunus* species

	*P. yedoensis*	*P. domestica*	*P. avium*	*P. dulcis*	*P. persica*	*P. yedoensis var. nudiflora*	Total
70%^a^							
Number of TNL single-gene	4	0	6	9	6	1	26
Number of TNL multi-gene	158	3	70	78	99	1	409
Proportion of TNL multi-gene (%)	97.53	100.00	92.11	89.66	94.29	50.00	94.02
Number of TNL gene family	30						
Average identity of TNL gene family (%)	87.44						
Number of non-TNL single-gene	31	30	38	28	15	12	154
Number of non-TNL multi-gene	396	328	170	166	198	99	1357
Proportion of non-TNL multi-gene (%)	92.74	91.62	81.73	85.57	92.96	89.19	89.81
Number of non-TNL gene family	103						
Average identity of non-TNL gene family (%)	88.27						
Number of multi-gene	554	331	240	244	297	100	1766
Proportion of multi-gene (%)	94.06	91.69	84.51	86.83	93.40	88.50	90.75
80%^b^							
Number of TNL single-gene	11	0	11	13	11	1	47
Number of TNL multi-gene	151	3	65	74	94	1	388
Proportion of TNL multi-gene (%)	93.21	100.00	85.53	85.06	89.52	50.00	89.20
Number of TNL gene family	55						
Average identity of TNL gene family (%)	88.97						
Number of non-TNL single-gene	64	62	64	38	27	21	276
Number of non-TNL multi-gene	363	296	144	156	186	90	1235
Proportion of non-TNL multi-gene (%)	85.01	82.68	69.23	80.41	87.32	81.08	81.73
Number of non-TNL gene family	158						
Average identity of non-TNL gene family (%)	89.94						
90%^c^							
Number of TNL single-gene	48	0	35	34	38	1	156
Number of TNL multi-gene	114	3	41	53	67	1	279
Proportion of TNL multi-gene (%)	70.37	100.00	53.95	60.92	63.81	50.00	64.14
Number of TNL gene family	75						
Average identity of TNL gene family (%)	94.51						
Number of non-TNL single-gene	138	150	124	84	68	48	612
Number of non-TNL multi-gene	289	208	84	110	145	63	899
Proportion of non-TNL multi-gene (%)	67.68	58.10	40.38	56.70	68.08	56.76	59.50
Number of non-TNL gene family	191						
Average identity of non-TNL gene family (%)	93.84						

^a^The coverage and identity values between sequences were both larger than 70%

^b^The coverage and identity values were larger than 80%

^c^The coverage and identity values were larger than 90%

For the TNL gene families, the proportions of multi-genes in four of the species were approximately 90%; specifically, 97.53% in *P. yedoensis*, 92.11% in *P. avium*, 89.66% in *P. dulcis*, and 94.29% in *P. persica*, except *P. domestica* had the highest proportion of TNL multi-genes (100%), and *P. yedoensis* var. *nudiflora* had the lowest proportion of 50% (Table 2). Similarly, for non-TNL genes, approximately 90% of the genes could be clustered into multi-gene families in four of the species: 92.74% in *P. yedoensis*, 91.62% in *P. domestica*, 92.96% in *P. persica*, and 89.19% in *P. yedoensis* var. n*udiflora*, except *P. avium* (81.73%) and *P. dulcis* (85.57%), possessed relatively lower proportions of non-TNL multi-genes. In addition, the proportion of TNL multi-genes was greater than that of non-TNL multi-genes in five of the *Prunus* species. The exception was *P. yedoensis* var. *nudiflora,* with 50% of TNL genes in multi-gene families and 89.19% of non-TNL genes in families (Table 2). Furthermore, the average identities within the TNL multi-gene families and the non-TNL multi-gene families were 87.44% and 88.27%, respectively (Table 2). The identity values significantly differed between TNL and non-TNL gene families, which indicated that the non-TNL gene families possessed significantly greater identity values than the TNL gene families (*t* test, *P* < 0.01; Table S1).

Subsequently, stricter criteria for coverage and identity (> 80% and > 90%) were used to further elucidate the relatively recent duplications of the NBS-LRR genes among the six species. When the coverage and identity thresholds were changed to > 80%, the proportions of TNL and non-TNL multi-genes decreased among the six *Prunus* genomes. The proportion of TNL multi-genes was still greater than 80% in the five species but was only 50% in *P. yedoensis* var. *nudiflora* (Table 2)*.* The proportion of non-TNL multi-genes exceeded 80% in five *Prunus* species, but *P. avium* displayed the lowest proportion of non-TNL multi-genes at 69.23% (Table 2). However, the proportions of multi-genes were significantly lower with the stricter criteria (> 80%) than with the criteria of > 70% in the six species (*t* test, *P* < 0.05).

When the cutoff values for coverage and identity were increased to the most stringent criteria (> 90%), the proportions of TNL multi-genes in the six species all exceeded 50%. For the TNL genes i*n P. domestica* under the three criteria (> 70%, > 80% and > 90%), the three TNL genes always belonged to the multi-genes, indicating that they might undergo recent and species-specific duplications. The proportion of non-TNL multi-genes still exceeded 50% in five species but was only 40.38% in *P. avium* (Table 2). The proportions of multi-genes were significantly lower in all six species using the strictest thresholds (> 90%) than when using standards of > 80% (*t* test, *P* < 0.01). Taken together, these results indicated that recent duplication events contributed to the expansion of NBS-LRR genes in the six *Prunus* species.

### NBS-LRR gene expansions in six *Prunus* species

*Ks* commonly represents the time elapsed since gene duplication events (Peterson and Masel 2009) and thus could be used to explore the duplication ages of the NBS-LRR genes in the six *Prunus* genomes. The *Ks* values were calculated for each TNL and non-TNL gene family according to the criteria of identity and coverage values of > 70%. Because of the influence of nucleotide substitution saturations, only *Ks* values below 1 were kept for further analysis in this study.

**Fig. 1:**
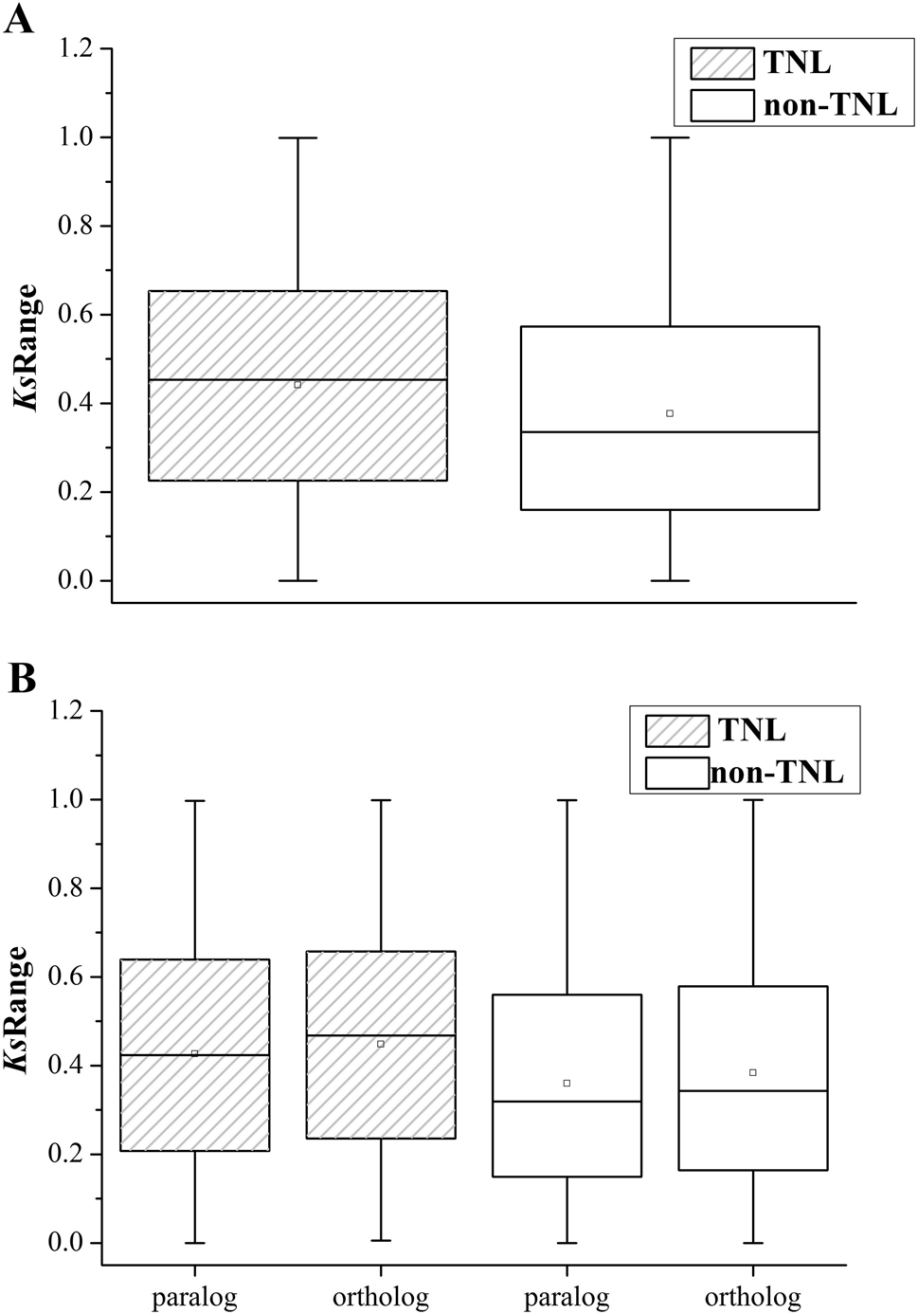
*Ks* values of NBS-LRR genes in six *Prunus* species. **A** The *Ks* values of TNLs and non-TNLs. **B** *Ks* values of paralogs and orthologs in TNLs and non-TNLs. The top and bottom bars are maximum and minimum values of the *Ks* ratios; the top and bottom of the box borders show third and first quartiles of the *Ks* ratios; the bar and small square in the box mean average and median values

TNL genes had higher median, first and third quartile and mean *Ks* values than non-TNL genes (Fig. 1A). A *t* test analysis also showed that the TNL genes had significantly larger *Ks* values than the non-TNL genes (*P* < 0.01). Moreover, there were two obvious peaks within the *Ks* distribution for the TNL gene paralogs, the frequencies of which were relatively similar. The higher peak was from 0.1 to 0.3, and the relatively lower peak was from 0.5 to 0.8. These two peaks represented two distinct expansion periods at a relatively ancient stage (0.5–0.8) and a relatively recent time (0.1–0.3) during the ongoing duplication events of the TNL genes. Within the *Ks* distribution for non-TNL gene paralogs, there was a more prominent peak, which ranged from 0.1 to 0.2. This peak was indicative of relatively young duplications that occurred in the non-TNL genes. In addition, a less conspicuous peak was located from 0.5 to 0.6 with a lower frequency. This peak suggested that a smaller-scale expansion occurred in a relatively ancient period (Fig. S1).

These results indicated that there were persistent occurrences of duplication events during the evolution of TNL and non-TNL genes among the six species. Recent duplications can play an important role in the expansion of both TNL and non-TNL genes. However, a higher proportion of TNL genes was generated by ancient duplications than by non-TNL genes. Furthermore, for both TNL and non-TNL genes, the *Ks* values of the orthologs were significantly higher than those of the paralogs (*t* test, *P* < 0.01; Fig. 1B). These results demonstrated that, in general, the species differentiation of the six *Prunus* species emerged earlier than the duplications of the NBS-LRR genes.

### Selective pressure on NBS-LRR genes in six *Prunus* species

The ratio of nonsynonymous to synonymous nucleotide substitutions (*Ka*/*Ks*) is an important parameter for detecting selective constraints on target genes (Zhong et al. 2018). A *Ka*/*Ks* ratio larger than 1 indicates positive selection on genes; if the ratio is equal to 1, this demonstrates neutral selection; and a *Ka*/*Ks* ratio less than 1 indicates purifying selection.

A fraction of the NBS-LRR genes had *Ka/Ks* values larger than 1, including 6.07% of the TNL gene pairs (512/8438) and 1.10% of the non-TNL gene pairs (314/28454). This indicated that positive selection was working on these genes in response to the rapid changes in the genomes of different pathogens. In contrast, the remaining TNL and non-TNL gene pairs had *Ka*/*Ks* ratios less than 1, illustrating that their evolution was driven by purifying selection. In addition, *Ka*/*Ks* values were approximately equal to 1 in seven TNL and 11 non-TNL gene pairs; these genes might face pseudogenization or no functionalization under neutral mutations.

**Fig. 2:**
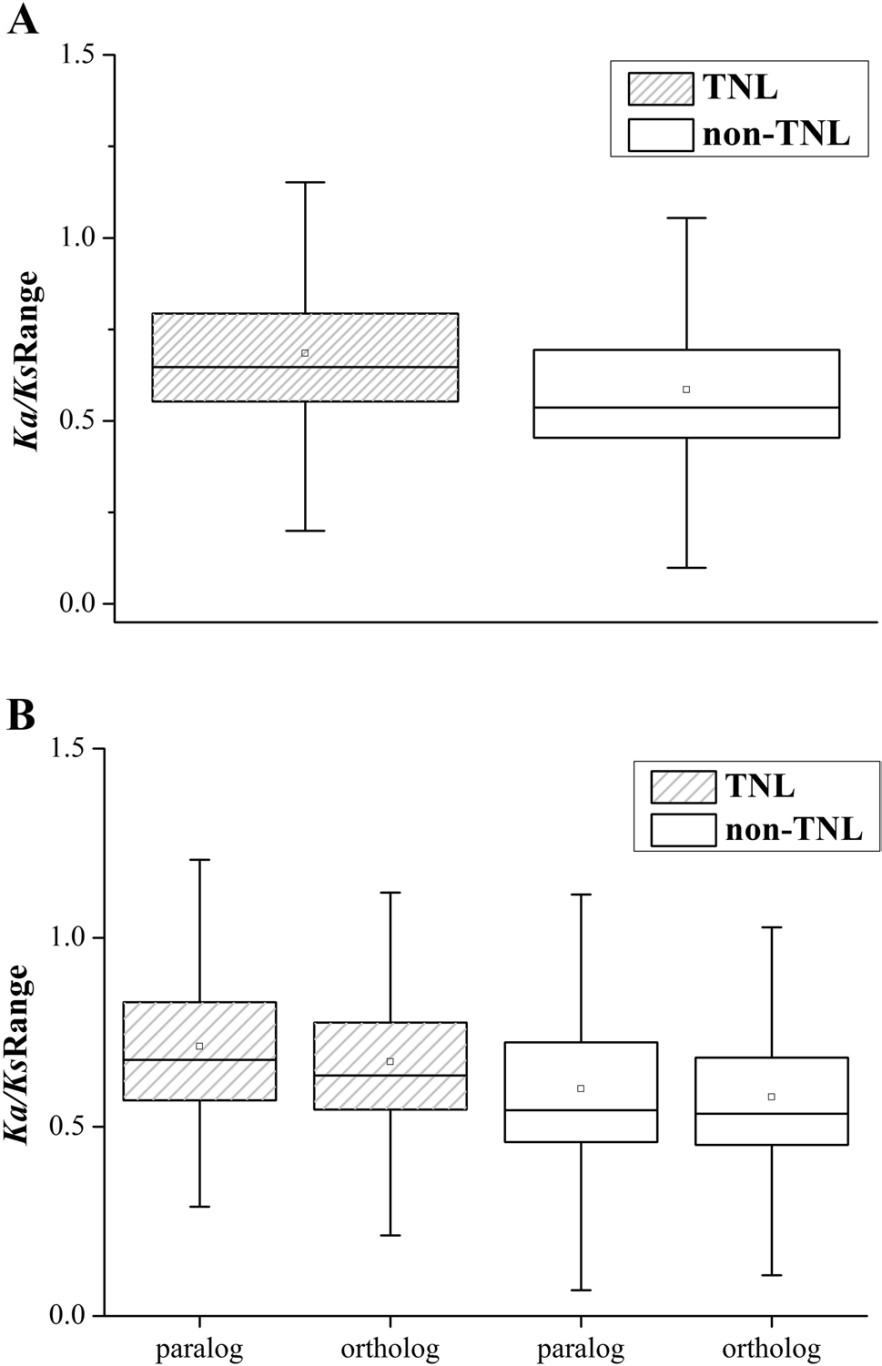
The *Ka/Ks* ratios of NBS-LRR genes in six *Prunus* species. **A** The *Ka/Ks* values of TNL and non-TNL genes. **B** The *Ka/Ks* values of paralogs and orthologs in TNL and non-TNL genes

The TNL genes had significantly larger *Ka*/*Ks* values than the non-TNL genes (*t* test, *P* < 0.01). This was supported by higher third and first quartile, median and average *Ka*/*Ks* ratio values of TNL genes than of non-TNL genes (Fig. 2A). This result indicated that the TNL genes might be under stronger selective pressure and experience more rapid evolutionary courses than the non-TNL genes. Moreover, there were significant differences between the *Ka*/*Ks* values of the paralogs and orthologs in both the TNL and non-TNL gene families (*t* test, *P* < 0.01; Fig. 2B). These differences demonstrated that NBS-LRR gene paralogs were subjected to weaker selection pressure in the six species.

**Fig. 3:**
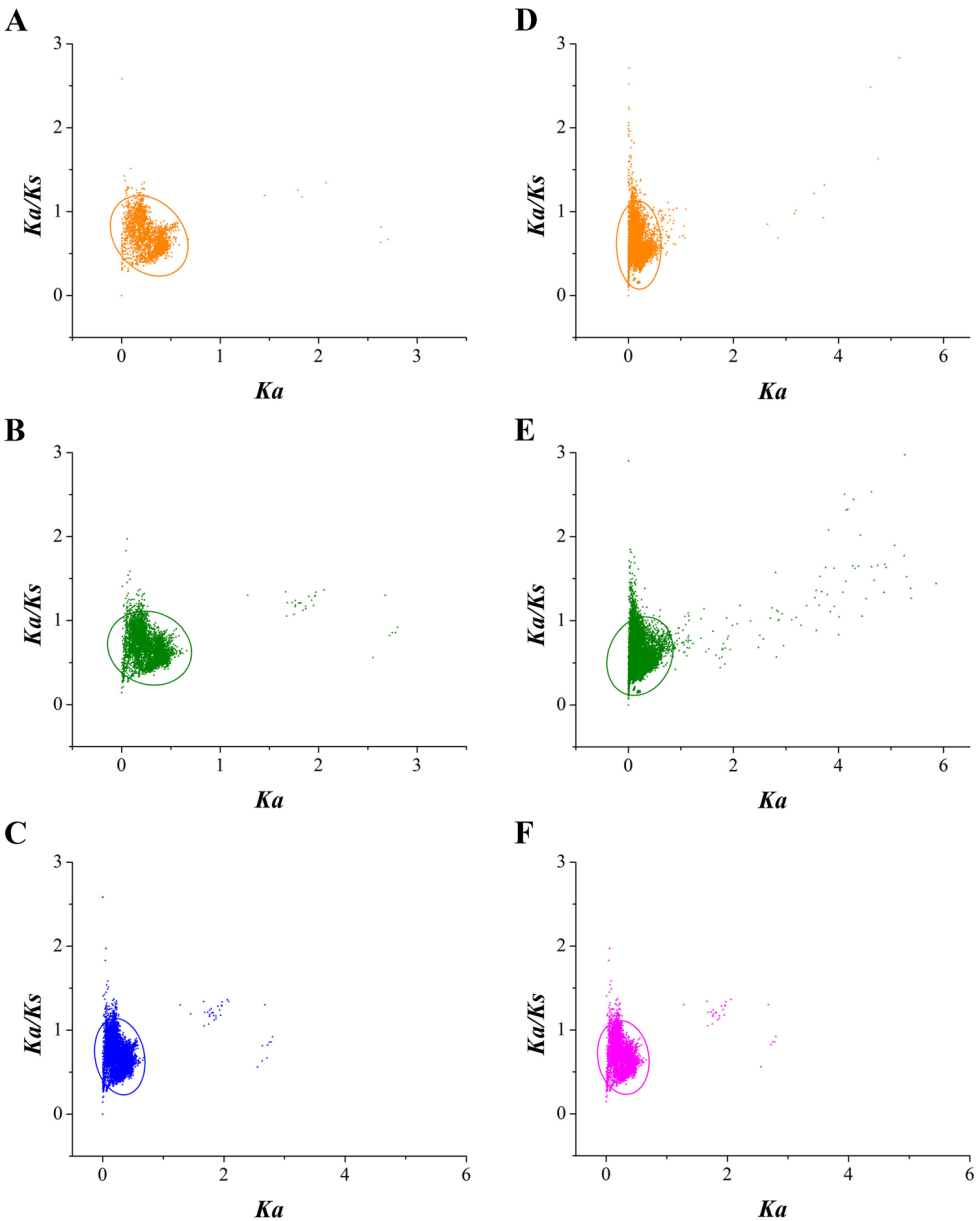
Distribution of *Ka* and *Ka/Ks* values of NBS-LRR genes in six *Prunus* species. The orange triangles represent the paralogs in TNL (**A**) and non-TNL multi-genes (**D**), and the green triangles represent the orthologs in TNL (**B**) and non-TNL multi-genes (**E**). The blue and purple triangles represent all TNL (**C**) and non-TNL multi-genes (**F**), respectively

A scatter matrix analysis was adopted to draw confidence ellipses of *Ka* and *Ka/Ks* values using the default confidence level of 95%. The relationships between the *Ka* and *Ka/Ks* values of TNLs and non-TNLs showed a broader distribution of *Ka* values for the non-TNLs than for the TNLs over the same *Ka/Ks* ratio ranges (Fig. 3C and F). Similarly, at the same *Ka/Ks* values, the *Ka* values of orthologs spread over a wider scope than those of paralogs in non-TNL gene families (Fig. 3D and E). For the relationships between *Ka* and *Ka/Ks* values of TNL genes, higher *Ka* values were observed for orthologs than for paralogs at the same *Ka/Ks* values (Fig. 3A and B), which illustrated that the paralogs possessed more functional conservation than the orthologs.

### Variation in NBS-LRR genes among six *Prunus* species

The nucleotide diversity (*Pi* value) represents the divergence distance between two genes in each gene family.

**Fig. 4:**
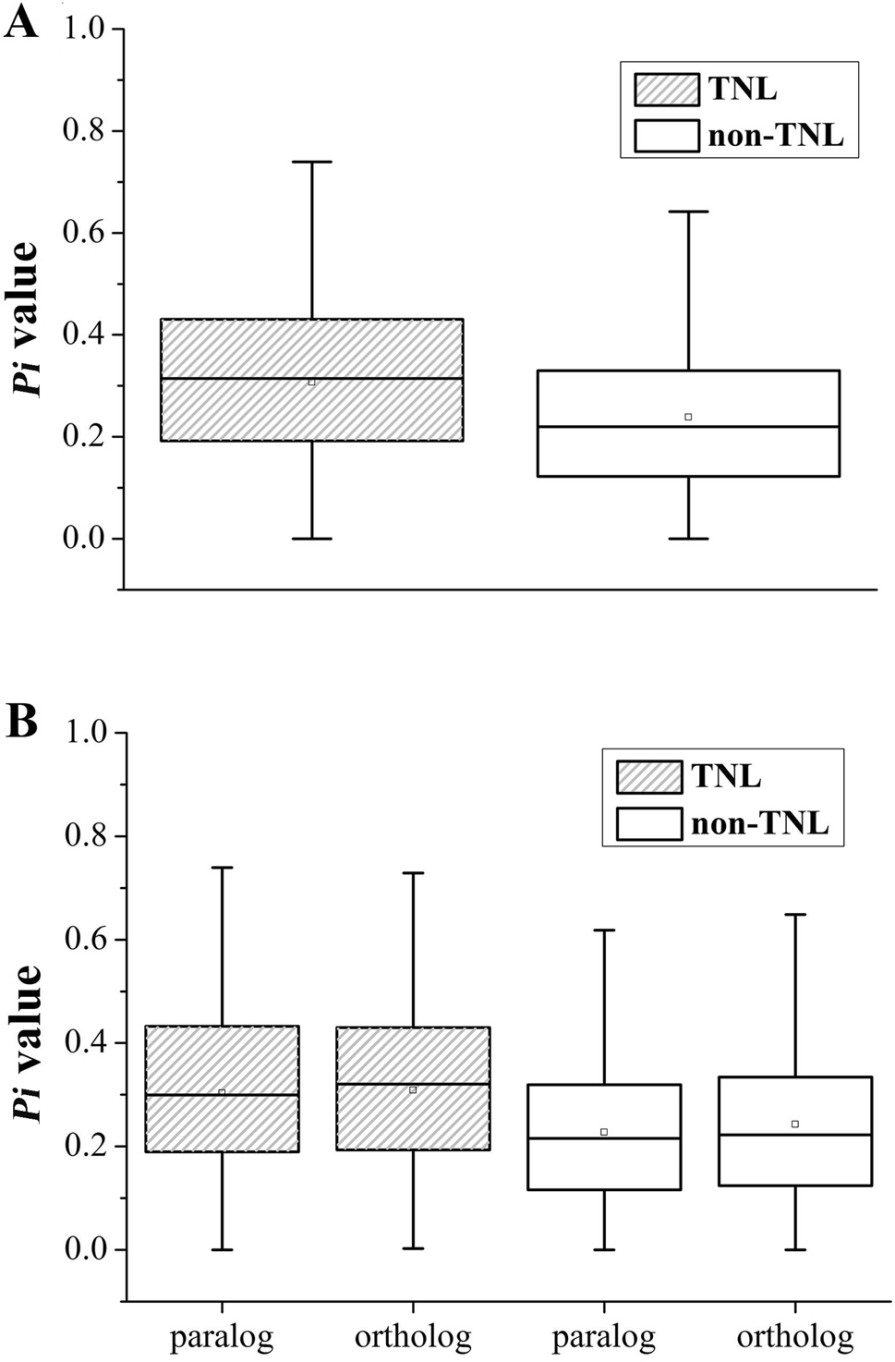
Nucleotide diversity (*Pi* values) of NBS-LRR genes in six *Prunus* species. **A** The *Pi* values of TNLs and non-TNLs. **B** The *Pi* values of paralogs and orthologs in TNLs and non-TNLs

In terms of the first and third quartiles, media and average values of the *Pi* values, the TNL genes were always higher than the non-TNL genes (Fig. 4A). In addition, *t* test analysis was carried out and revealed that the *Pi* values of TNL genes were significantly higher than those of non-TNL genes (*P* < 0.01). In addition, the orthologs had slightly higher *Pi* values than paralogs in TNL genes. Moreover, a *t* test analysis showed that the *Pi* values of orthologs were significantly greater than those of paralogs in non-TNL gene families (*P* < 0.01; Fig. 4B).

A total of 1604 sequence exchange events were identified in NBS-LRR gene families, among which more exchange events were detected in non-TNLs (1244) than in TNLs (360). Moreover, orthologs (247) had higher sequence exchange events than paralogs (113) in TNL genes. The sequence exchange events of orthologs (849) were significantly greater than those of paralogs (395) in non-TNLs (*t* test, *P* < 0.05; Table S2), speculating that the frequent sequence exchange between the orthologs might contribute to the diversification of non-TNL genes.

### Phylogenetic analysis of NBS-LRR genes in six *Prunus* species

**Fig. 5:**
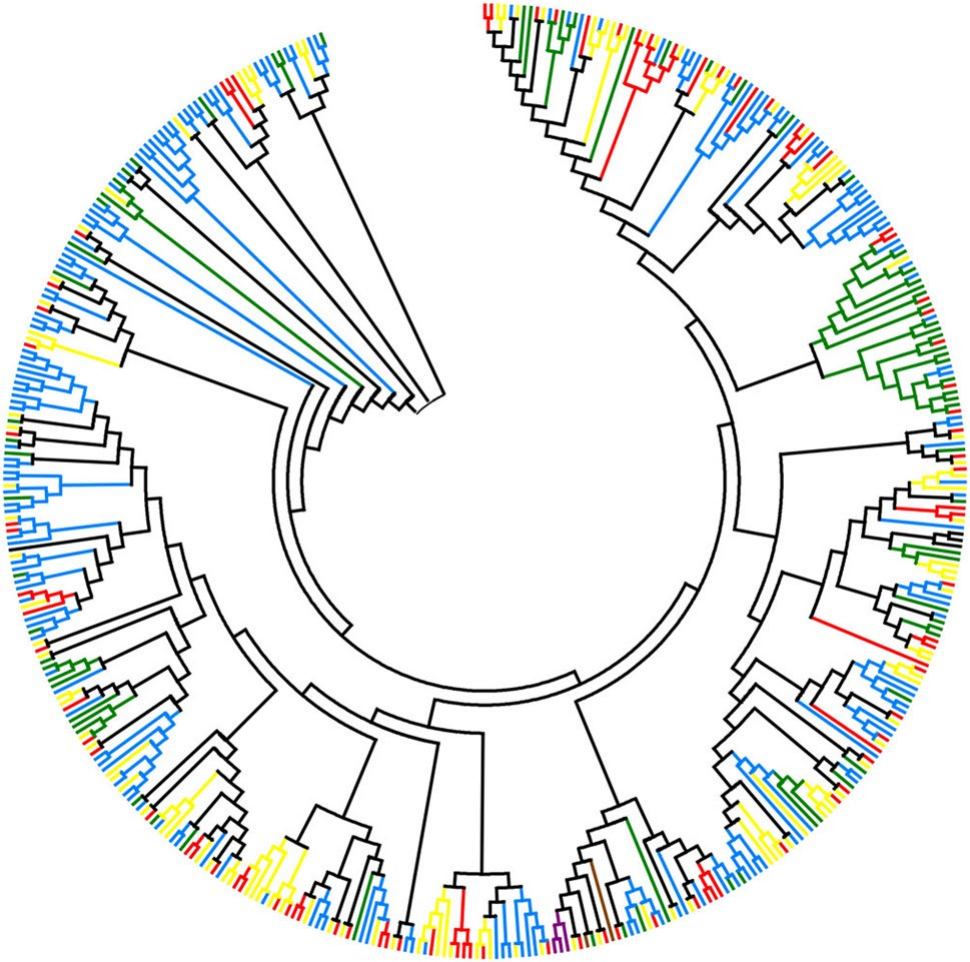
Phylogenetic tree of TNL genes among six *Prunus* species. The blue, purple, green, red, yellow, and brown branches represent TNL genes from *P. yedoensis*, *P. domestica*, *P. axium*, *P. dulcis*, *P. persica* and *P. yedoensis* var*. nudiflora*, respectively

**Fig. 6:**
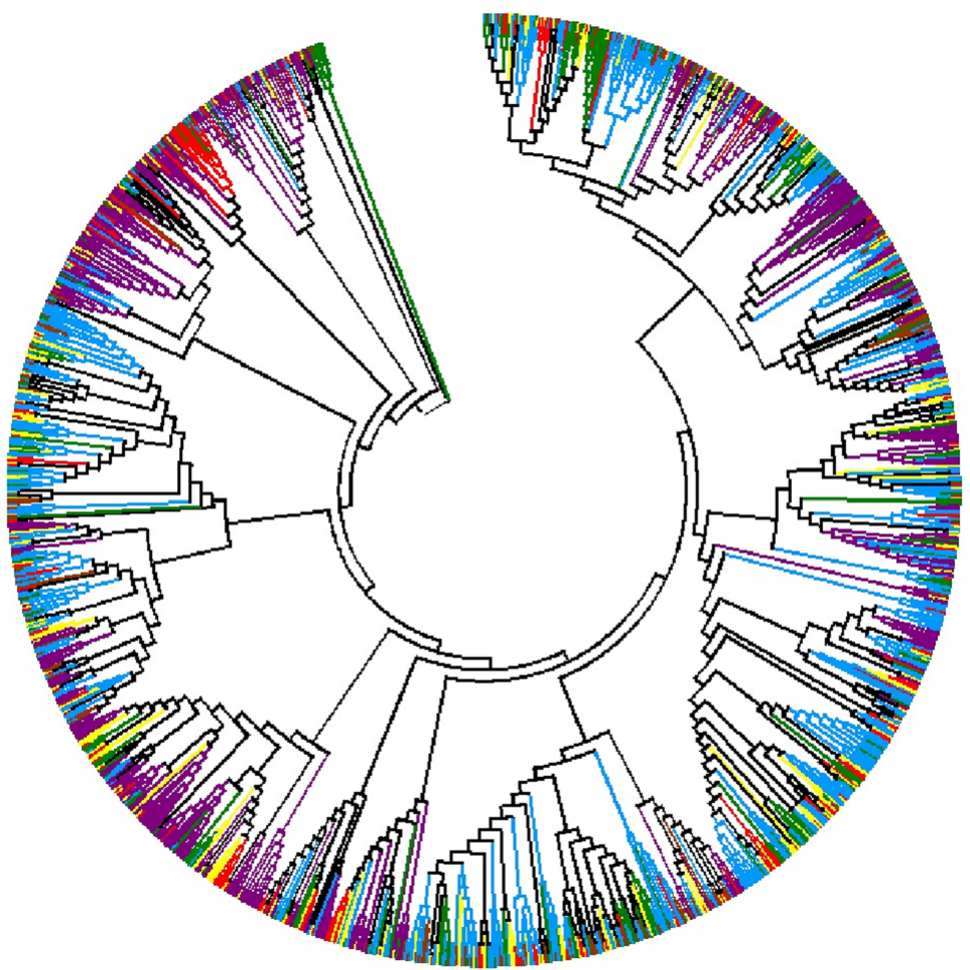
Phylogenetic tree of non-TNL genes among six *Prunus* species. The blue, purple, green, red, yellow, and brown branches represent non-TNL genes from *P. yedoensis*, *P. domestica*, *P. axium*, *P. dulcis*, *P. persica* and *P. yedoensis* var*. nudiflora*, respectively

Phylogenetic trees were constructed based on the nucleotide sequences of the NBS domains of the TNL (Fig. 5) and non-TNL (Fig. 6) genes. Within the two phylogenetic ML trees, two types of clades could be defined using bootstrap values larger than 50, including species-specific and lineage-specific duplicated clades. The species-specific duplicated clades, which are represented by the vertical blue lines, include NBS-LRR genes for which duplications only appeared in one *Prunus* species (Fig. S2). The lineage-specific duplicated clades, represented with vertical red lines, encompass gene duplications that occurred in two or more *Prunus* species (Fig. S3).

The TNL phylogenetic tree displayed 44 species-specific duplicated clades, including 99 NBS-LRR genes involved in species-specific duplications (Fig. S2). Interestingly, the highest number of TNL genes related to species-specific duplications was 51 in *P. yedoensis*. Two of the three TNL genes in *P. domestica* clustered together in a species-specific duplicated clade, which might indicate gene contraction of the TNLs in this *Prunus* species. However, no TNL genes were found in species-specific duplicated clades of *P. yedoensis* var. *nudiflora*. The results showed that 22.76% (99/435) of the TNL genes were generated by species-specific duplications, demonstrating that species-specific duplications were partially responsible for the expansions of TNL genes among five of the *Prunus* species (all species except *P. yedoensis* var. *nudiflora*). In addition, there were 17 lineage-specific duplicated clades in the TNL tree, consisting of 100 genes, showing that 22.99% (100/435) of the TNL genes were produced by lineage-specific duplications. This indicated that lineage-specific duplications also played a partial role in TNL gene expansions.

Compared with the TNL tree, the non-TNL tree contained more species-specific duplicated clades (181) and lineage-specific duplicated clades (29; Fig. S3). There were 432 non-TNL genes in species-specific duplicated clades, illustrating that 28.59% (432/1511) of the non-TNL genes were generated from species-specific duplications among the six *Prunus* species. Only 13.83% of the non-TNL genes (209/1511) were discovered in lineage-specific duplicated clades. Moreover, 22 RPW8-NBS-LRR (RNL) genes were located in the relatively basal clade of the non-TNL tree (Fig. 6). Among the species-specific duplicated non-TNL genes, nine RNL genes were discovered in four species-specific duplicated clades (Fig. S3). Therefore, both species-specific duplications and lineage-specific duplications partially contributed to the expansions of the NBS-LRR genes among the six *Prunus* species (Fig. 5 and 6).

### DEGs of NBS-LRRs in peaches R36 and S38 after aphid infestation

To verify the disease-resistance function of the NBS-LRR genes, we performed differential expression analysis of the NBS-LRR genes in *P. persica* infected by aphids. According to the RNA-seq data of two peach genotypes infected by aphids (Niu et al. 2018a), 48 NBS-LRR genes were found to exhibit differential expression among all NBS-LRR genes of *P. persica* among R36 and S38.

**Fig. 7:**
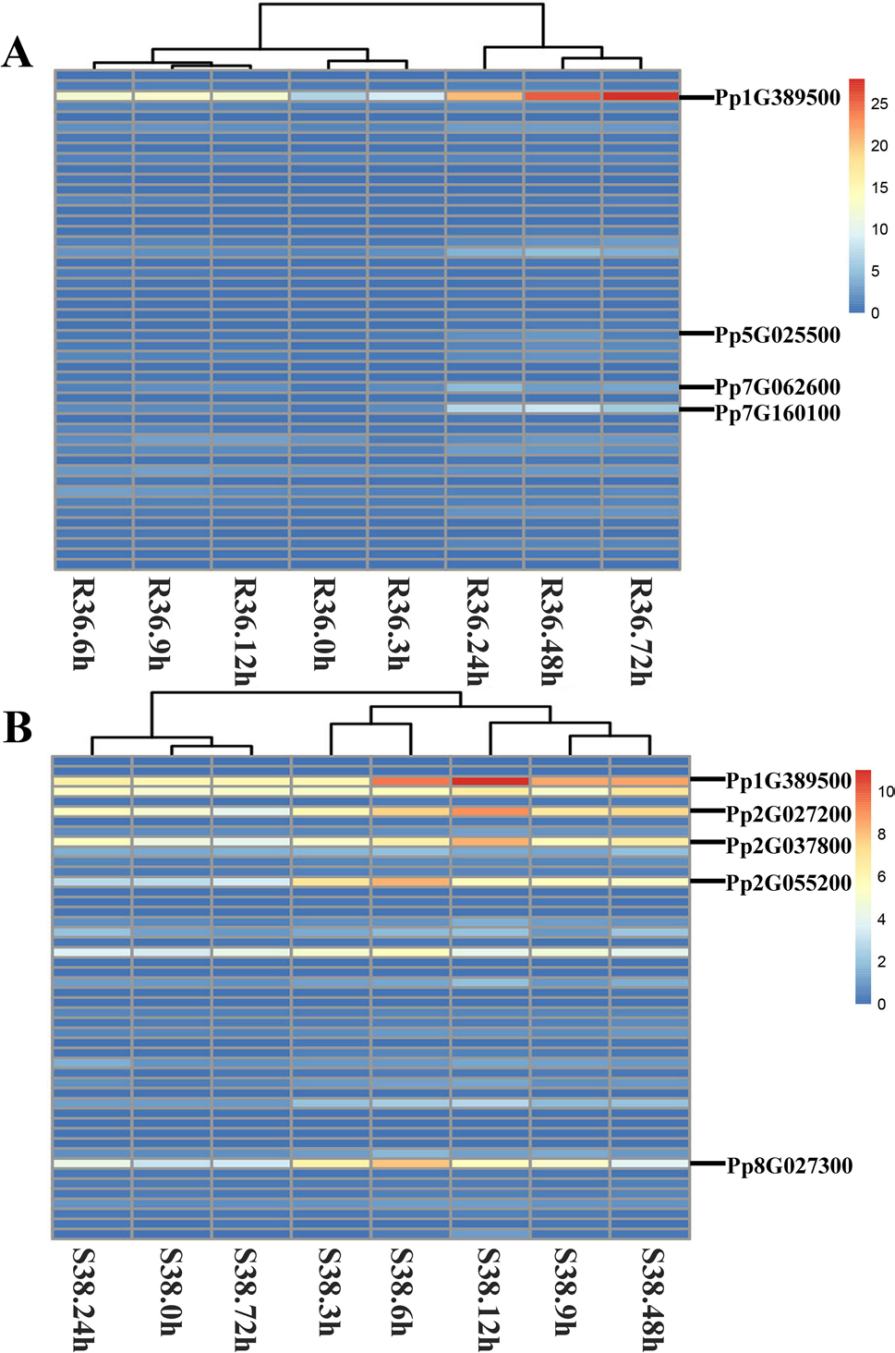
Expression heatmaps of DEGs of NBS-LRRs in *P. persica* R36 and S38 treated with aphid infection. **A** and **B** represent the resistant genotype R36 and the susceptible genotype S38, respectively. The h means hour after the infection treatment. The eight NBS-LRR genes were marked as representative DEGs in response to aphid infection

There were different expression patterns of the NBS-LRR genes in R36 and S38 based on hierarchical clustering analysis (Fig. 7). For the resistance genotype R36, the time points of 0, 3, 6, 9, and 12 h clustered in a close group, and the later periods of infection (24, 48, and 72 h) were in another group. That is, the expression levels presented a general increasing trend with the infection time, and the differentially expressed NBS-LRR genes reached their peak from 24 to 72 h after infestation in R36 leaves. For example, the expression levels of Pp5G025500, Pp7G062600 and Pp7G160100 slowly increased from 0 to 36 h but decreased slightly at 72 h. Interestingly, the Pp1G389500 gene had a relatively high expression level at 0 h, was basically steadily upregulated from 0 to 72 h, and reached its expression peak at 72 h. However, the differences in expression mode in susceptible genotype S38 were that the infection time points of 0, 24 and 72 h clustered together, and the relatively early periods of 3, 6, 9, 12, and 48 h clustered in another class. These results indicated a general waved regulation tendency of NBS-LRR expression; in other words, obvious upregulation and downregulation appeared alternately during the whole infection process in S38. The maximum levels of many differentially expressed NBS-LRRs were observed at 6 h or 12 h (Fig. 7B). Pp1G389500, Pp2G027200 and Pp2G037800 are representative examples of the waved expression pattern in S38. They first upregulated from 0 to 6 h, after a slight decrease at 9 h, and then peaked at 12 h; subsequently, they underwent downregulation, upregulation and downregulation from 12 to 72 h. In addition, another two genes, Pp2G055200 and Pp8G027300, also possessed waved expression levels, but their highest expression quantities were found at 6 h.

Therefore, the different expression patterns between R36 and S38 resulted in relatively lower average expression levels at the early time points of 0 to 12 h in R36 than in S38. However, the average expression levels at the later time points from 24 to 72 h were larger in the resistant genotype R36 than in the susceptible genotype S38. One notable exception was the gene Pp1G389500, whose expression quantities were significantly larger in R36 than in S38 (*t* test, *P* < 0.05). Taken together, the results demonstrated that the resistant strain R36 had a longer defense duration than the susceptible strain S38.

According to the expression level analysis, eight representative DEGs of NBS-LRR genes were screened out, including 2 TNLs and 6 non-TNLs. After locating their positions on the two phylogenetic trees and conforming their *Ka/Ks* values with other family members, one TNL gene (p2G055200) was found to possess a *Ka/Ks* value larger than 1 and was located with a paralog in a clade with relatively short branch lengths in the phylogenetic tree (Fig. S4). Taken together, it could be inferred that this gene might undergo a relatively fast evolutionary rate and might be a candidate gene for disease-resistance breeding in peaches.

## Discussion

In this study, 1946 NBS-LRR genes were detected in the six *Prunus* plant genomes. Based on the multi-gene classification and *Ks* distributions, the NBS-LRR genes revealed that large-scale expansions occurred at recent and relatively ancient stages. Interestingly, these NBS-LRR gene expansions were collectively driven by species-specific and lineage-specific duplications among the six *Prunus* species. In addition, the TNL and non-TNL genes experienced different duplication times and selective pressures, demonstrating that the two subtype NBS-LRR genes might respond to distinct *Avr* genes with different variation rates. These results supported that the NBS-LRR genes of the six *Prunus* species were shaped by different genetic events in conflict with specific pathogens in the environments, which could be considered a resistance gene pool for *Prunus* plant breeding.

### Expansions of NBS-LRR genes occurred at different periods

Gene duplication plays an active and crucial role in the enhancement of genetic diversity and the emergence of novel functions in organisms (van der Hoorn and Kamoun 2008). In plants, large numbers of duplicated genes are produced by ancient or recent gene duplications and are then retained for environmental adaptation and growth and development requirements (Panchy et al. 2016). For example, the disease-resistance NBS-LRR genes experience duplications on different scales and at different time points across plant species (van der Hoorn and Kamoun 2008). In the model plant *A. thaliana*, a total of 149 NBS-LRR genes have been created by widespread duplications and some small-scale genetic events (Meyers et al. 2003). For Rosaceae species, approximately half of the NBS-LRR genes have been derived from recent duplications in apple and pear genomes (Zhong et al. 2015). In this study, although the six *Prunus* species were derived from a common ancestor, the absolute numbers of NBS-LRR genes differed. The Yoshino cherry (*P. yedoensis*) genome had a high number of NBS-LRR genes, which was approximately 5.21-, 2.07-, and 1.85-times greater than the number of NBS-LRR genes in *P. yedoensis* var. *nudiflora*, *P. avium* and *P. persica*, respectively. The distinct variations in NBS-LRR gene number might be due to more excessively large predicted genes in the whole-genome sequences of *P. yedoensis* than in those of the other species.

The classification of multi-gene families reflected the periods of NBS-LRR gene duplications. The results indicated that duplications mainly emerged in more recent times (recent duplications or young duplications). Previous studies have stated that recent duplications have led to wide arrays of NBS-LRR- and NBS-encoding multi-genes in the genomes of grapevine, poplar, kiwi fruit (*Actinidia chinensis*), apple, pear, and mei (Yang et al. 2008; Zhong et al. 2015; Li et al. 2016). In the present study, for the strictest standards of coverage and identity values (> 90%), there were significantly lower percentages of TNL and non-TNL multi-genes than when using the undemanding standards of > 70% and > 80% (*t* test, *P* < 0.01). Nevertheless, more than 50% of the NBS-LRR genes were incorporated into multi-gene families in the investigated species under the criteria of > 90%. An exception to this was that only 40.38% of non-TNL genes were classified into families in *P. avium* (Table 2). These results clearly showed that approximately half of the NBS-LRR genes were generated via recent duplications in the six *Prunus* species.

In addition to the multi-gene classification, the distribution of the *Ks* values of paralogs highlighted periods of continuous duplication of the NBS-LRR genes across the six species. During the uninterrupted duplications of the NBS-LRR genes, two expansion peaks with similar *Ks* ranges were detected in the TNL (0.1–0.3) and non-TNL gene families (0.1–0.2; Fig. S1). The smaller *Ks* values may demonstrate that recent duplications contributed to the expansions of these related genes after the speciation of the six *Prunus* plants. As ligneous plants with long life cycles, *Prunus* species are likely to be challenged by various pathogens during their growth, development, and aging stages. Similarly, the concentrations at smaller *Ks* ratios in other ligneous plants, such as poplar, have previously been reported to manifest the importance of recent duplication events in the evolution of the NBS-LRR genes (Yang et al. 2008). The genetic mechanism underlying the influence of recent duplications on the NBS-LRR genes might be that the duplications provide a large material library for heritable variations, and the specific host plants can use them to resist species-specific pathogens (Parniske et al. 1997; Yang et al. 2008; Zhong et al. 2015).

Nevertheless, relatively ancient duplications were also discovered underlying the NBS-LRR genes of the six species. These ancient duplications were indicated by the other two peaks at relatively large values within the *Ks* distributions for TNL gene paralogs (0.5–0.8) and non-TNL gene paralogs (0.5–0.6; Fig. S1). It could be inferred that these NBS-LRR gene expansions emerged before the speciation of the six plants. After a variety of genetic events, the offspring copies after a variety of genetic events were reserved to provide defense against lineage-specific bacterial and viral pathogens or insects.

### Species-specific and lineage-specific duplications jointly contributed to NBS-LRR gene expansions

According to the gene duplications emerging in one or more species, species-specific duplications and lineage-specific duplications of the NBS-LRR genes could be elucidated in the six *Prunus* species. Species-specific duplications represent gene duplications that occur in one species in response to selective pressure exerted by certain species-specific pathogens (Yang et al. 2008). Lineage-specific duplications represent gene duplications that occur in two or more species with close genetic relationships (Cannon et al. 2002). In this study, according to the TNL gene phylogenetic tree, 22.76% and 22.99% of the TNL genes were associated with species-specific and lineage-specific duplications, respectively. However, for the non-TNL genes, more species-specific duplicated genes (28.59%) were found than lineage-specific duplicated genes (13.83%). Therefore, both species-specific and lineage-specific duplications participated in the evolutionary processes of NBS-LRR genes. Species-specific duplications contributed more strongly to non-TNL gene expansions than lineage-specific duplications in the six *Prunus* species. Combining the results of the phylogenetic tree and *Ks* value analyses, it could be deduced that the species-specific duplications may correspond to the recent duplications (*Ks* peaks at 0.1–0.2) that occurred after the speciation of the six *Prunus* species. Furthermore, the lineage-specific duplications showed a great correlation with the relatively ancient duplications (*Ks* peaks at 0.5–0.8 in TNL and 0.5–0.6 in non-TNL) that arose before *Prunus* diversification.

The inference could be examined (not shown in Results) and further consolidated on the basis of the previously estimated mutation rate for peach, which was 9.48 × 10^–9^ point mutations per site per generation (Xie et al. 2016). Considering that the species studied were from the same genus, this same mutation rate could be used in combination with the two paralog *Ks* peaks to estimate the expansion times of the NBS-LRR genes. Recent species-specific duplications were roughly predicted 31.65–63.29 million years ago (MYA), and relatively ancient lineage-specific duplications began over 158.23 MYA. The recent duplications thus coincided with the *Prunus* speciation that occurred at approximately 36–44 MYA (Baek et al. 2018) and represented species-specific duplications. Relatively ancient duplications occurred before the appearance of the *Prunus* genus approximately 61–88 MYA (Chin et al. 2014; Baek et al. 2018) and represented lineage-specific duplications. Therefore, both the species-specific duplications and the lineage-specific duplications, by means of NBS-LRR gene expansions, were constantly in conflict with specific pathogens driven by various environmental factors in the six *Prunus* species.

### The two subclasses of NBS-LRR genes evolved differently among the six Prunus species

The two subclasses of NBS-LRR genes, TNLs and non-TNLs, can be traced back to the ancient period of green algae. Since then, TNLs and non-TNLs have exhibited different copy number variations and evolutionary rates and have been subjected to different selective pressures (Shao et al. 2016, 2019). In this study, significantly fewer TNL gene members were found than non-TNL gene members (*t* test,* P* < 0.05) across the plant genomes. This could be explained by the fact that TNL genes were exposed to an extended period of gene contraction, and CNLs, the main essential component of non-TNLs, experienced gradual gene expansions during the stages after angiosperm divergence at approximately 100–225 MYA (Shao et al. 2016). In addition to copy number differences, distinct evolutionary speeds between the two subclasses have also frequently been reported in plants. For example, TNL genes were found to have significantly larger *Ks* values than non-TNL genes in *Arabidopsis*, *Fragaria* and soybean species (Chen et al. 2010; Zhang et al. 2011; Zhong et al. 2015, 2018). In the present study, as expected, the TNL genes possessed significantly greater *Ks* values than the non-TNL genes (*t* test, *P* < 0.01). Broadly speaking, the TNL genes duplicated earlier than the non-TNL genes in the six *Prunus* species.

Through further analysis of the *Ks* ranges, it could be deduced that a larger percentage of TNL genes were anciently duplicated than non-TNL genes, which would explain the *Ks* differences between the two subclasses. These results illustrated that more TNL genes were manufactured by relatively ancient duplications than non-TNL genes, and TNL genes were shaped more rapidly than non-TNL genes for environmental adaptation. Furthermore, it has been found that stronger selection pressures work on TNL genes than on non-TNL genes in the investigated genomes, Rosaceae plants and soybean plants (Zhang et al. 2011; Zhong et al. 2015, 2018). This could be deduced by the significantly greater *Ka/Ks* values for TNL genes than for non-TNL genes. The arms race between plants and pathogens leads to long-term coevolutionary histories between plant *R* genes and pathogenic *Avr* genes (Petit-Houdenot and Fudal 2017). Consequently, the diverse evolutionary profiles of the TNLs and non-TNLs might be due to the coevolution of corresponding *Avr* genes along with faster or slower rates of genetic variation.

Moreover, the *Pi* values of TNLs were greater than those of non-TNL gene families, revealing higher sequence divergence between TNL genes than non-TNLs. These results also demonstrated the different evolution patterns between TNL and non-TNL genes, in which the TNL genes evolved faster than non-TNLs (Yang et al. 2008; Chen et al. 2010). The sequence exchange events of non-TNLs were greater than those of TNLs, which might contribute to accumulating more variations for creating novel *R* genes in six *Prunus* species (Chen et al. 2010).

Within the non-TNL subclass, the RNL genes (which include RPW8-CNL and RPW8-XNL here) have been regarded as an independent subgroup of NBS-LRR genes for the past few years. The RNL genes also have an ancient origin; the rapid emergence and divergence of TNLs, CNLs, and RNLs can be traced back to the stage prior to green algae divergence (Shao et al. 2019). Accordingly, the RNL genes always cluster into an independent clade distributed in the basal nodes of the phylogenetic tree and can be easily distinguished from the TNLs and CNLs. This has been shown in potato and legume genomes (Jupe et al. 2012; Shao et al. 2014, 2016). This was also the case for the RNL genes of the genomes investigated in the present study (Fig. 6), which further verifies their ancient origin and their sister relationship with CNL genes in the six *Prunus* species.

### NBS-LRR genes have different expression patterns in response to aphid infection between resistant and susceptible* P. persica*

Previous studies have shown that DEGs mainly play a role in perception, signal transduction, secondary metabolism, transcriptional regulation and plant–pathogen interactions (Gervasi et al. 2018; Ma et al. 2020; Wan et al. 2021). Among the two genotypes of flax (*Linum usitatissimium* L.), the upregulated genes in the resistant cultivar to *Fusarium oxysporum* were significantly higher than those in the susceptible cultivar (Dmitriev et al. 2017). Similarly, after bananas (*Musa spp*.) were infected with *F. oxysporum*, more DEGs had higher expression levels detected in resistant cultivars ‘Yueyoukang 1’ than in susceptible cultivars ‘Baxijiao’ (Niu et al. 2018b). These results were consistent with those in *P. sogdiana*, in which the expression level of the *R*-gene PsoRPM2 in resistant plants was significantly higher than that in susceptible plants after infection with root-knot nematodes (RKNs) (Zhu et al. 2017). In the present study, the differentially expressed NBS-LRR genes from the two peach lines R36 and S38 had distinct responses to aphid infection, showing that the NBS-LRR genes from the resistant genotype had relatively longer defense durations than those from the susceptible genotype. Surprisingly, a similar phenomenon was also previously reported in two peach cultivars, and more DEGs were involved in the response to *Xanthomonas arboricola* pv. *pruni* in the susceptible cultivar ‘JH Hale’ at the early infection period, while the resistant cultivar ‘Redkist’ had a greater number of DEGs during the later period (Gervasi et al. 2018).

## Supplementary Information

Below is the link to the electronic supplementary material.

Supplementary file1 Fig. S1. Ks ranges of NBS-LRR genes among six Prunus species (TIF 2487 KB)

Supplementary file2 Fig. S2. Phylogenetic tree of TNL genes among six Prunus species. (TIF 26823 KB)

Supplementary file3 Fig. S3. Phylogenetic tree of non-TNL genes among six Prunus species. (TIF 77080 KB)

Supplementary file4 Fig. S4. The clade of the TNL phylogenetic tree containing the p2G055200 gene. The gene p2G055200 is marked by red highlight. (TIF 522 KB)

Supplementary file5 (XLSX 415 KB)

Supplementary file6 (XLSX 15 KB)

Supplementary file7 (XLSX 22 KB)

Supplementary file8 (XLSX 36 KB)

## Declarations

### Research involved with human or animals participants

This article does not contain any studies with human participants or animals performed by any of the authors.
