# Supplementary figures and images for: Different scales of gene duplications occurring at different times have jointly shaped the NBS-LRR genes in Prunus species
Source: Mol Genet Genomics. 2022 Jan 15;297(1):263–76. doi: 10.1007/s00438-021-01849-z (PMC8803762; doi:10.1007/s00438-021-01849-z)

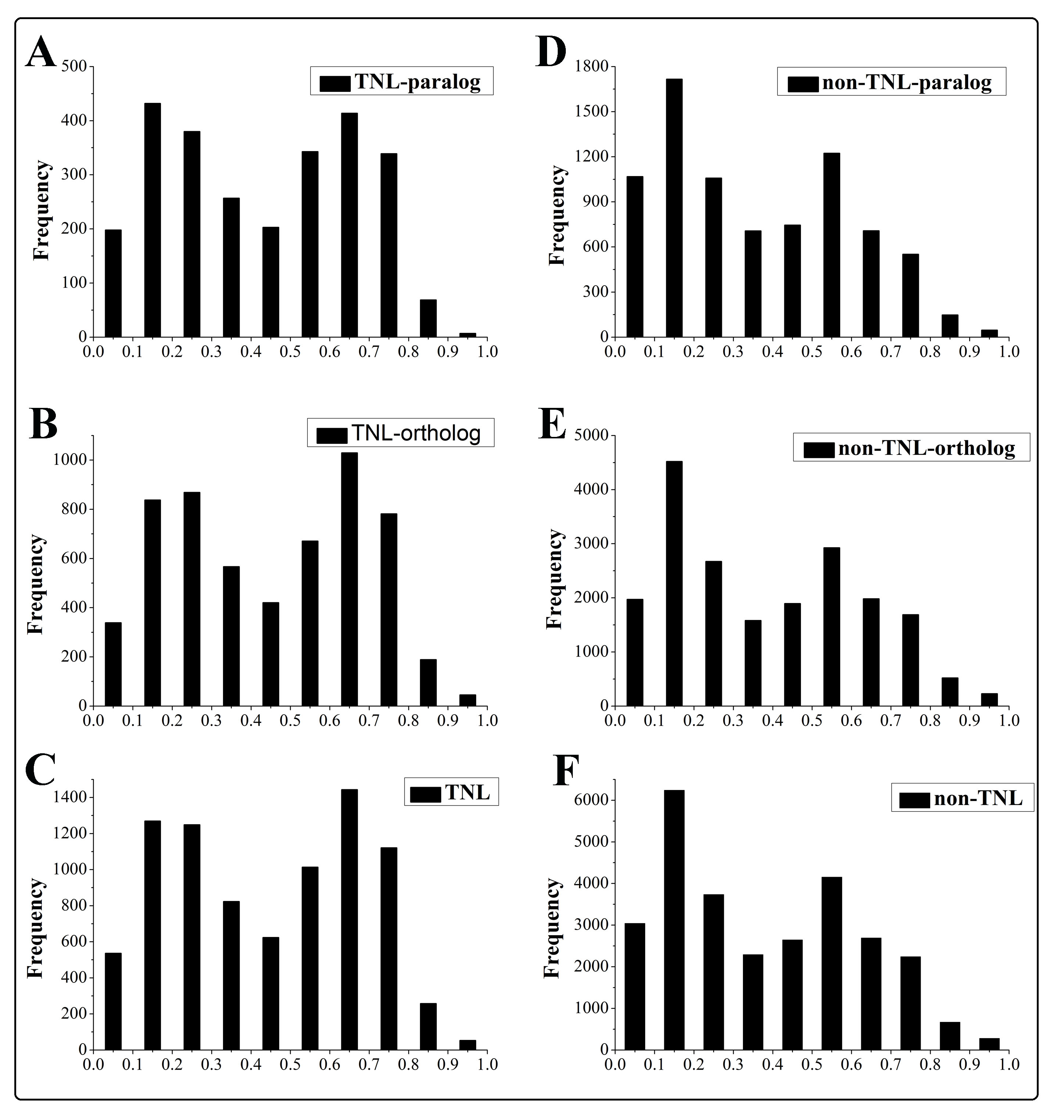

Supplement: Supplementary file 1 — Supplementary file1 Fig. S1. Ks ranges of NBS-LRR genes among six Prunus species (TIF 2487 KB) [file 438_2021_1849_MOESM1_ESM.tif]

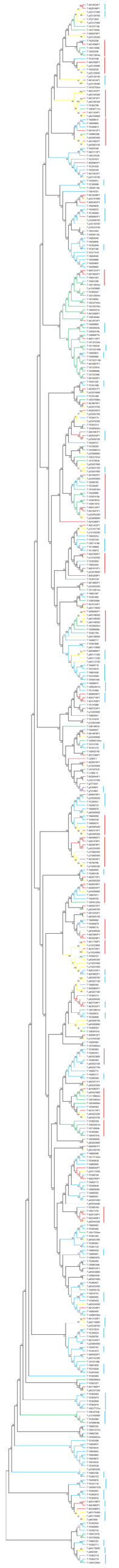

Supplement: Supplementary file 2 — Supplementary file2 Fig. S2. Phylogenetic tree of TNL genes among six Prunus species. (TIF 26823 KB) [file 438_2021_1849_MOESM2_ESM.tif]

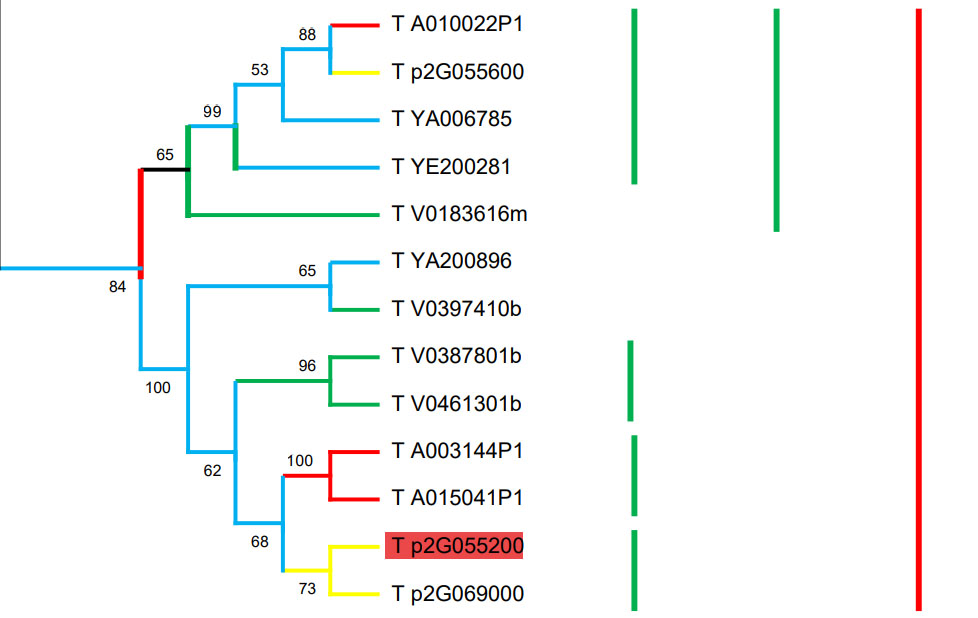

Supplement: Supplementary file 4 — Supplementary file4 Fig. S4. The clade of the TNL phylogenetic tree containing the p2G055200 gene. The gene p2G055200 is marked by red highlight. (TIF 522 KB) [file 438_2021_1849_MOESM4_ESM.tif]
